# Transcriptome Analysis Reveals the Effect of Long Intergenic Noncoding RNAs on Pig Muscle Growth and Fat Deposition

**DOI:** 10.1155/2019/2951427

**Published:** 2019-06-25

**Authors:** Guoting Chen, Xiaofang Cheng, Gaoli Shi, Cheng Zou, Lin Chen, Jingxuan Li, Mengxun Li, Chengchi Fang, Changchun Li

**Affiliations:** ^1^Key Laboratory of Agricultural Animal Genetics, Breeding and Reproduction of the Ministry of Education and Key Laboratory of Swine Genetics and Breeding of the Ministry of Agriculture, Huazhong Agricultural University, Wuhan, China; ^2^The Cooperative Innovation Center for Sustainable Pig Production, Wuhan, China

## Abstract

Muscle growth and fat deposition are the two important biological processes in the development of pigs which are closely related to the pig production performance. Long intergenic noncoding RNAs (lincRNAs), with lack of coding potential and the length of at least 200nt, have been extensively studied to play important roles in many biological processes. However, the importance and molecular regulation mechanism of lincRNAs in the process of muscle growth and fat deposition in pigs are still to be further studied comprehensively. In our study, we used the data, including liver, abdominal fat, and longissimus dorsi muscle of 240 days' age of two F2 full-sib female individuals from the white Duroc and Erhualian crossbreed, to identify 581 putative lincRNAs associated with pig muscle growth and fat deposition. The 581 putative lincRNAs shared many common features with other mammalian lincRNAs, such as fewer exons, lower expression levels, and shorter transcript lengths. Cross-tissue comparisons showed that many transcripts were tissue-specific and were involved in the important biological processes in their corresponding tissues. Gene ontology and pathway analysis revealed that many potential target genes (PTGs) of putative lincRNAs were involved in pig muscle growth and fat deposition-related processes, including muscle cell proliferation, lipid metabolism, and fatty acid degradation. In Quantitative Trait Locus (QTLs) analysis, some PTGs were screened from putative lincRNAs, MRPL12 is associated with muscle growth, GCGR and SLC25A10 were associated with fat deposition, and PPP3CA, DPYD, and FGGY were related not only to muscle growth but also to fat deposition. Therefore, it implied that these lincRNAs might participate in the biological processes related to muscle growth or fat deposition through homeostatic regulation of PTGs, but the detailed molecular regulatory mechanisms still needed to be further explored. This study lays the molecular foundation for the in-depth study of the role of lincRNAs in the pig muscle growth and fat deposition and further provides the new molecular markers for understanding the complex biological mechanisms of pig muscle growth and fat deposition.

## 1. Introduction

Muscle growth and fat deposition are complex quantitative traits and important economic traits in pig production. Muscle growth directly affects the meat quality and fat deposition of pigs that seriously affect pork quality and production efficiency. To a large extent, the two factors affect the consumer choices for pork [1–3]. In recent years, people have begun to pay more attention to pork quality rather than quantity. Duroc, a typical Western lean pig breed, has good characteristics of fast growth, high lean meat percentage, and high feed utilization rate [4]. Now it is widely used in commercial production. By contrary, Erhualian, a typical Chinese native dual-purpose pig breed, shows a good characteristic of high intramuscular fat content, which is delicate and juicy for processed foods [5]. Using these two pig breeds as parents, the F2 generations produced show different extreme phenotypes. Therefore, analyses of extreme performance individuals of the F2 generation provide a good opportunity to explore the molecular mechanism of muscle growth and fat deposition in these two breeds of pigs and further provide the new ideas for breeding pigs with better meat taste.

lincRNAs are a type of intergenic transcripts that are longer than 200bp in length and have almost no protein-coding ability [6–8]. Compared with protein-coding transcripts, lincRNAs have shorter transcript length, fewer exons number, stronger tissue specificity, and lower conservation [7, 9, 10]. So far, many lincRNAs have been discovered and identified for their corresponding functions. According to reports, there are 15,512 human lincRNAs and more than 10,000 mouse lincRNAs have been identified and some of them have been shown to play important roles in many biological processes [11–13], such as gene regulation [14, 15], stem cell pluripotency [16, 17], X chromosome inactivation [18], and skeletal muscle development [7, 19]. However, compared with humans and mouse, many lincRNAs in pigs are still uncharacterized. The relationship between some lincRNAs and their potential target genes (PTGs) remains unclear too; meanwhile, there are relatively little understanding of the effects of lincRNAs on muscle growth and fat deposition.

In our experiment, we performed RNA-seq data analysis on the transcripts of liver, abdominal fat, and longissimus dorsi muscle in the F2 full-sib pig individuals of Duroc and Erhualian pigs by using the data that have been published on the NCBI. A total of 581 putative lincRNAs were identified and the basic features of these lincRNAs were also characterized. We obtained differentially expressed lincRNAs (DELs) from different tissues of two pigs by differential expression analysis. By conducting gene ontology and pathway analysis of the PTGs of lincRNAs, we found that some DELs were significantly involved in the regulation of muscle growth and fat deposition-related biological processes. Through QTLs analysis of DELs, it was shown that most of the DELs could positively regulate their PTGs expression. This result provides directions and goals for further studies on molecular mechanism underling pig muscle growth and fat deposition; meanwhile, it also provides valuable data for the modern biotechnology to breed new pig breeds with the good characteristics of fast growth as well as high intramuscular fat deposition.

## 2. Materials and Methods

### 2.1. Ethics Statement and Data Acquisition

The experimental protocols in our study were approved by the Ethics Committee of Huazhong Agricultural University in 2013 (HZAUMU2013-0005), and all the data were downloaded from the NCBI SRA website with the accession number provided by Wang et al. (Table 1). These datasets samples contained 6 RNA-seq samples, including liver, abdominal fat, and longissimus dorsi muscle of 240 days' age of two F2 full-sib female individuals from the white Duroc and Erhualian crossbreed. Each of the 6 RNA-seq samples has 3 technical replicates, and the 3 samples are randomly taken from different parts of the same sample for sequencing. Then the technically repeated data are combined for subsequent analysis [20]. The pig gene annotations files were downloaded from http://ftp.ensemblorg.ebi.ac.uk/pub/release-91/gtf/sus_scrofa/, and the human and mouse lincRNAs references data were downloaded from http://asia.ensembl.org/info/data/ftp/index.html.

### 2.2. RNA-Seq Reads Mapping and Transcriptome Assembly

The quality of sequencing reads was evaluated by Fastqc command. The raw reads were filtered and trimed by Trimmomatic (version 0.36) with default parameters [21]. Then, clean reads were mapped to the pig reference genome (Sus scrofa 11.1) using HISAT2 (version 2.0.2) [22] with default parameters. The mapped reads were sorted and removed duplicates by SAMtools (version 0.1.19) [23]. Usually, StringTie (version 1.2.2) [22] was used to assemble the mapped reads with default parameters, and we set the “−G” option of StringTie for novel transcript assembly. Then, we used StringTie to merge the 6 assembled transcript files (GTF format) of tree groups into a nonredundant transcriptome.

### 2.3. Pipeline for lincRNA Identification

**Figure 1:**
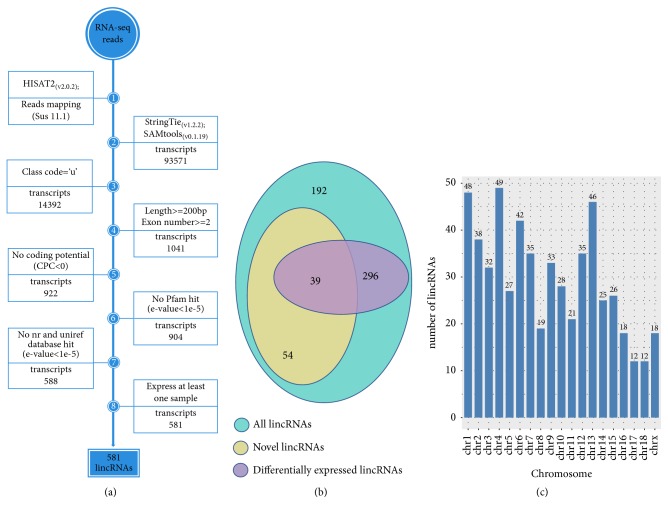
(a) Integrative pipeline for the identification of putative lincRNAs in this study. (b) Venn diagram of all lincRNAs and novel lincRNAs and differentially lincRNAs. (c) The chromosome distribution of putative lincRNAs.

We processed nonredundant transcriptome through the lincRNAs detection pipeline to identify the lincRNAs, and the main steps are as follows (Figure 1): (1) filter the transcripts with ‘U' category categorized (represent intergenic transcripts) by gffcompare program. (2) Reserve transcripts with exon >1 and length >200 bp. (3) The coding potential of the transcripts in both strands can be calculated by a coding potential calculator (CPC) tool [24], and the transcripts with cpc>0 were removed. (4) In order to evaluate whether the remaining transcripts contain any known protein-coding domain, the HMMER-3 was used to identify the transcripts translated in all six possible frames with homologs that were concluded in any of the known protein family domain in the Pfam database, and transcripts that matched to the Pfam hit (E-value < 1 × 10–5) were excluded. (5) The transcripts with the similarity to the known protein in against the NCBINR and UniRef90 database [25] would be filtered out by the BLASTX program with an E-value < 1 × 10–5. (6) Reserve the transcripts expressed in at least one sample [22, 26].

### 2.4. Differential Expression lincRNAs Analysis

We used “htseq-count” program to count the number of reads across six samples from three tissues, and then, in order to identify the effects of different lincRNAs from different tissues on muscle growth and fat deposition, we used the “DESeq2” package to screen out the differential lincRNAs between any two tissues [27]. In the screening results, transcripts with log⁡2 (FoldChange) greater than 1 or less than -1, and the corrected padj value less than 0.05, are considered to be the differential lincRNAs in these two groups [28].

### 2.5. Comparisons between lincRNAs and Protein-Coding Transcripts

We selected the transcripts annotated as “protein-coding” in gene annotation file, and the obtained lincRNAs were screened with “known” and “novel” by “blastn” command. Then we compared the lincRNAs (“known” and “novel”) with these protein-coding transcripts in the following aspects: transcript length, exon number and length, expression level, and FPKM.

### 2.6. Screen the Adjacent Genes of lincRNAs

Many of the lincRNAs are unknown, and the annotations are not comprehensive. To determine the relevant functions of linRNAs, first of all, functional prediction is required, and then further functional verification is performed. At present, the main prediction methods are cis-type prediction (adjacent gene method) and trans-type prediction (correlation prediction) [29, 30]. It has been reported that lincRNAs may have a greater regulatory effect on adjacent genes. In mammals, lincRNAs can regulate some biological processes such as development and transcriptional regulation through its adjacent target genes (<10kb) (e.g., GATA2, GZF1, and NEUROG2) and then participate in relevant biological processes [31]. Therefore, in our study, we used adjacent gene method to predict the function of lincRNAs. BEDTools (version 2.17.0) can be used to screen the adjacent gene (10k or 100k) of each lincRNAs locus. By analyzing the functions of their adjacent genes (such as pathway analysis and QTLs analysis), we could predict the function of corresponding lincRNAs [32]. In order to make the results better, in the process of late functional prediction, the selected adjacent genes needed to be expressed in at least one sample.

### 2.7. Gene Ontology and Pathway Analysis

In order to query each protein-coding gene and understand their functions, the DAVID database was used to perform gene function enrichment analysis via Gene Ontology (GO) and Kyoto Encyclopedia of Genes and Genomes (KEGG) pathway enrichment analysis. And GO terms or KEGG pathways with corrected P-value less than 0.05 were considered to be enriched clusters. Due to the limitation of genes annotation in Sus scrofa, the BIOMART module in Ensembl was used to convert all genes into human homologues genes suitable for DAVID analysis [6].

### 2.8. QTLs Analysis of Differentially Expressed lincRNAs

To further predict the functions of all 581 putative lincRNAs, we selected 272 DELs from these putative lincRNAs to perform the QTLs analysis. The pig quantitative trait loci (QTLs) database was downloaded from the Animal QTLdb (Pig QTLdb) and the download path is https://www.animalgenome.org/cgi-bin/QTLdb/SS/download?file=bedSS_11.1. When doing QTL analysis, the main command was the “intersectBed” [33]. The p-value and Pearson correlation coefficient between each pair of lincRNAs and its PTGs obtained in RNA-seq were calculated with cor.test() function in the R software. When p-value < 0.05 [34, 35], it is considered to be statistically significant, and when the Pearson correlation coefficient is closer to 1, it indicates that the correlation between lincRNAs and its PTGs is higher [36].

### 2.9. Correlation Validation between lincRNAs and PTGs

We verified the quantitative relationship between lincRNAs and PTGs and selected 11 RNA samples from 3 tissues, including liver, abdominal fat, and longissimus dorsi muscle. For quantitative verification, total RNA was extracted using Trizol reagent (Invitrogen, Life Technologies, CA, USA) and performed according to the manufacturer's instructions. After checking the quality of RNA samples, cDNA synthesis for lincRNAs and PTGs detection was performed using the RevertAid First Strand cDNA Synthesis Kit (Thermo, Wuhan, Cat# k1622). According to the manufacturer's instructions, qPCR for lincRNAs and PTGs detection in Roche LightCyler 480 system (Roche, Mannheinm, Germany) was performed using SYBR Green (CWBIO, Beijing, China, CW0957). The qPCR data were analyzed using the 2-∆∆CT method and R scripts were used to perform related linear regression analysis.

## 3. Results

### 3.1. Transcripts Assembly and lincRNAs Identification

In order to identify and analyze lincRNAs associated with pig muscle growth and fat deposition, RNA-seq data involving three tissues of white Duroc and Erhualian F2 full-sib pig individuals were obtained from a previously published study and the main process of identifying lincRNAs was shown in Figure 1(a).

After removing the unqualified reads in raw reads with the “trimmomatic” command, approximately 217.2 of 225.5 million clean reads were obtained and mapped to the pig reference genome (Sus scrofa 11.1) by HISAT2 (Table 1). Then, we assembled the transcriptome for each sample (liver, abdominal fat, and longissimus dorsi muscle) by StringTie and synthesized all the transcripts into nonredundant transcripts by using StringTie-Merge program. After merging the nonredundant transcripts, about 15.38% (14,392 of 93,571) of the transcripts were defined as intergenic transcripts. The 581 putative lincRNAs encoded by 500 gene loci of 14,392 intergenic transcripts were obtained using the selection conditions shown in Figure 1(a), and the annotation files of all lincRNAs were shown in File S1. As all the samples were from female pigs, these 500 gene loci were not distributed on the Y chromosome and the detailed distribution map was shown in Figure 1(c). However, 93 of these 581 lincRNAs were novel lincRNAs (Figure 1(b)).

### 3.2. Characterization of Identified lincRNAs

**Figure 2:**
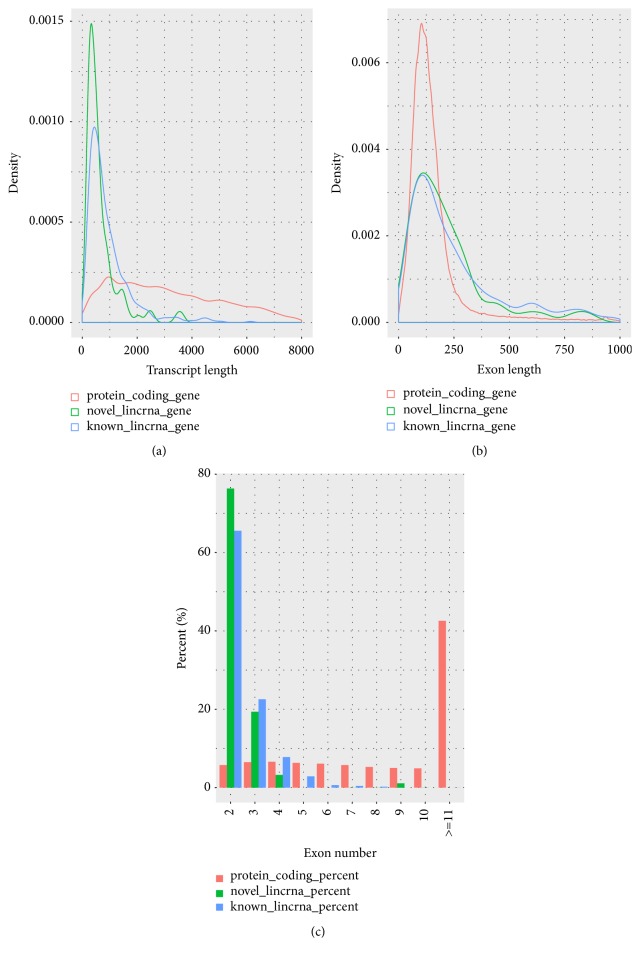
Characteristics of putative lincRNAs (compare with protein-coding gene). (a) Comparison of transcript length distribution. (b) Comparison of exon length distribution. (c) Comparison of exon number.

There are many differences between protein-coding transcripts and lincRNAs, such as the length of transcripts, exon number, and exon length. To verify these differences, we performed the comparison analysis. In this process, the putative lincRNAs (including novel lincRNAs and known lincRNAs) were compared with the protein-coding transcripts, and the characteristics of novel lincRNAs were analyzed during the comparison process. We obtained 45,788 protein-encoding transcripts, including 22,342 annotated genes in the Ensembl pig sequence database by reconstructing transcripts. In addition, the pig lincRNAs annotation file contains 12,103 known lincRNAs transcripts that corresponded to 7,381 lincRNAs genes. After identification, we found that the average transcripts lengths of protein-coding, novel lincRNAs, and known lincRNAs were 3285bp, 669bp, and 953bp, respectively. And the average transcripts length of novel lincRNAs was shorter than the other two transcripts (Figure 2(a)). In addition, the average exon lengths of the protein-coding transcripts, novel lincRNAs, and known lincRNAs were 283, 286, and 376, respectively. And it shows that the exon length of novel lincRNAs is shorter than that of known lincRNAs but longer than that of protein-coding transcripts (Figure 2(b)). Meanwhile, the average exon number of the protein-coding transcripts, novel lincRNAs, and known lincRNAs are 11.6, 2.3, and 2.5, suggesting that the average number of exons is approximately the same as that of known lincRNAs but much smaller than that of protein-coding transcripts (Figure 2(c)). The comparison results (shorter transcript length, smaller exon number, and longer exon length) of novel lincRNAs with protein-coding genes are basically consistent with previous reports [37, 38].

### 3.3. Expression Analysis of lincRNAs

**Figure 3:**
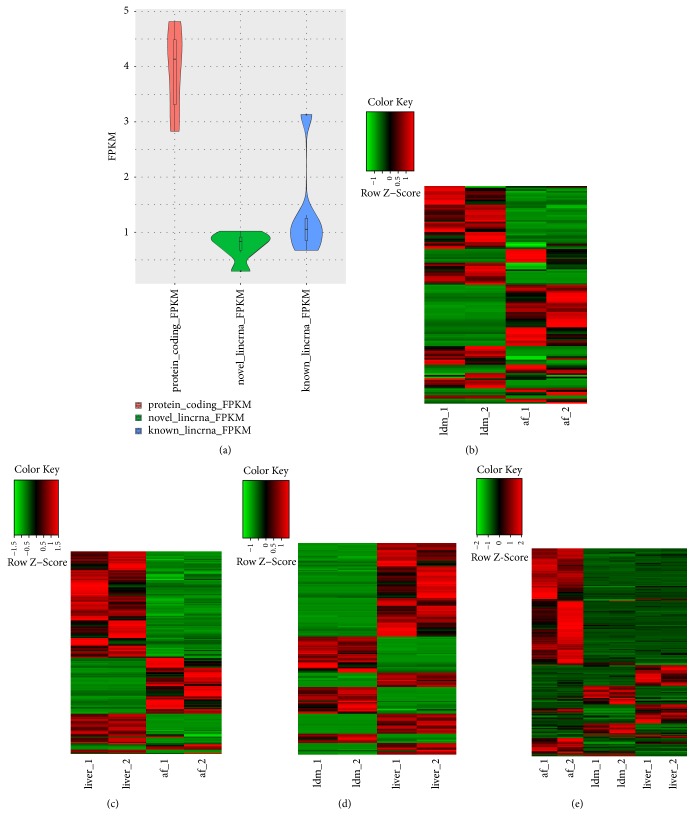
Expression profile of lincRNAs. (a) Comparison of expression level between lincRNAs (known and novel) and protein-coding genes. The curve indicates density distribution. (b) Differential lincRNAs expression heat map in abdominal fat* vs.* longissimus dorsi muscle group. (c) Differential lincRNAs expression heat map in abdominal fat* vs.* liver group. (d) Differential lincRNAs expression heat map in longissimus dorsi muscle* vs.* liver group. (e) Expression heat map of differentially expressed protein-coding genes in all tissues.

Previous reports showed that lincRNAs had lower expression level when compared with protein-coding transcripts [39]. To test this conclusion, we compared the average expression levels of the 581 putative lincRNAs (488 known lincRNAs and 93 novel lincRNAs) with the protein-coding transcripts to explore the lincRNAs expression profile. We found that putative lincRNAs (novel lincRNAs* vs.* known lincRNAs, 0.76FPKM* vs.* 1.34FPKM) showed indeed significantly lower expression level than that of protein-coding transcripts (3.95FPKM) (Figure 3(a)). Then, in order to identify the effects of differentially expressed lincRNAs from different tissues on muscle growth and fat deposition, we compared the three tissues (abdominal fat* vs.* longissimus dorsi (af* vs.* ldm), abdominal fat vs liver (af* vs.* liver), and longissimus dorsi muscle vs. liver (ldm* vs.* liver)) through the “DEseq2” package in the R software to get the differentially expressed genes (DEGs) between tissues. Finally, we obtained the numbers of the DEGs between different tissues which were 128 (af* vs.* ldm), 185 (af* vs.* liver), and 181(ldm* vs.* liver), respectively (Figures 3(b)–3(d)), and the specific expression of these DEGs between different tissues was shown in Tables S1–S3. There are more differentially expressed lincRNAs between different tissues of the same individual, and the lincRNAs expressed in the same tissue of different individuals have little difference (Figures 3(b)–3(d)). Meanwhile, 271 differentially expressed protein-coding genes were also identified (Figure 3(e)).

### 3.4. Nearest Neighbor Analysis of lincRNAs

**Figure 4:**
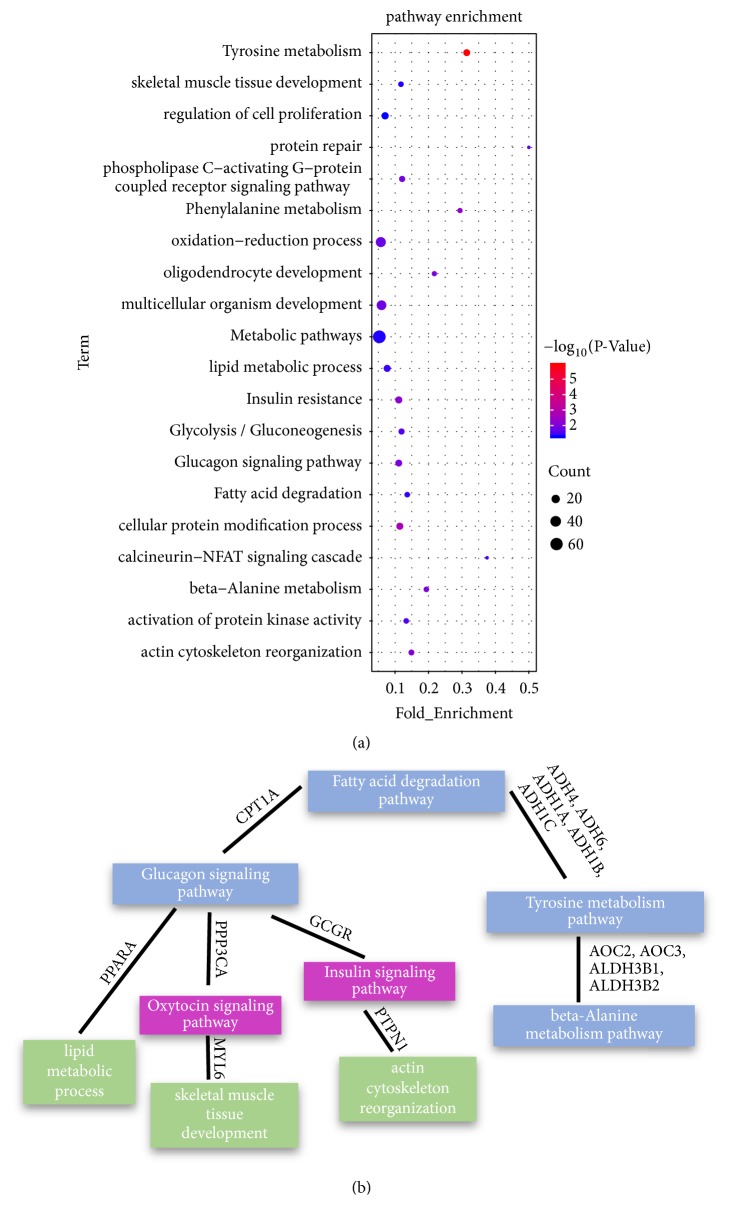
(a) Gene ontology and pathway analysis related to muscle growth and fat deposition of the potential target genes (PTGs) of differentially expressed lincRNAs (DELs). (b) Among the gene ontology and pathway analysis that are enriched, there are several major links between pathways involved in muscle growth and fat deposition. The blue module represents kegg pathway analysis and the green module represents gene ontology. The target gene between the two modules represents that this target gene coexists in the two pathways. For example, CPT1A exists in both glucagon signaling pathway and fatty acid degradation pathway, and most of these target genes are related to muscle growth or fat deposition.

Previous studies indicate that lincRNAs may act in a cis-manner to regulate the expression levels of their neighboring genes [40]. It is valuable to predict the corresponding function of lincRNAs by identifying the function of a protein-coding gene transcribed near lincRNAs (<100 kb). To predict the functional characteristics of putative lincRNAs, first of all, we obtained the adjacent protein-coding genes (<100 kb) of all 272 differentially expressed genes (DEGs) (lincRNAs) (Table S4). And then, we queried each protein-coding gene in the DAVID database and performed related pathway analysis [11] (Table S5). The results of the DAVID analysis showed that 216 of the 272 DEGs were significantly involved in 33 biological processes and 15 pathways. DELs had 147 nearby protein-coding genes involved in biological processes and pathways involved in muscle growth and fat deposition, such as skeletal muscle tissue development, actin cytoskeleton reorganization, lipid metabolic process, beta-alanine metabolism, fatty acid degradation, and glucagon and signaling pathway (Figure 4(a)). Interestingly, there are some links between these major pathways involved in muscle growth and fat deposition and several bridge genes among these pathways such as CPTIA, GCGR, PPP3CA, and PPARA. Some genes in the ADH and AOC family genes are related to the biological processes of muscle growth or fat deposition (Figure 4(b)).

### 3.5. QTLs Analysis of lincRNAs and Functional Prediction

**Figure 5:**
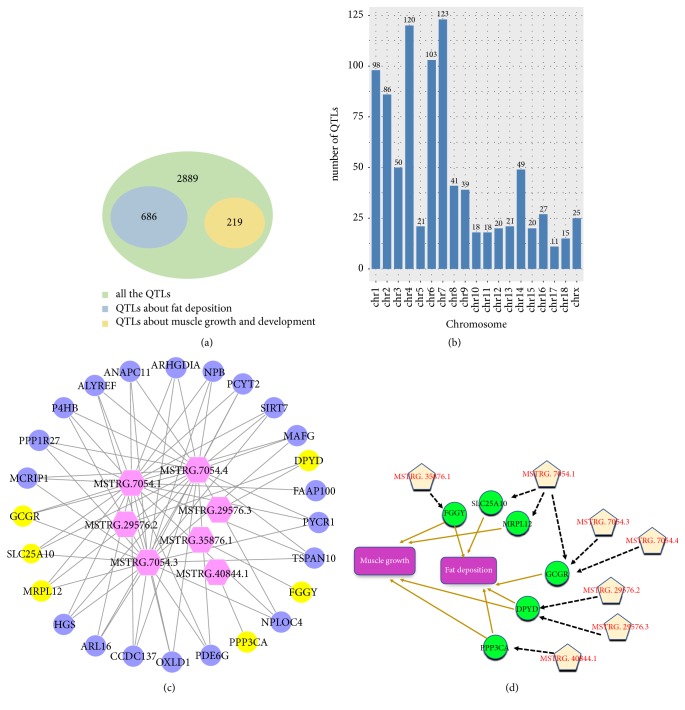
(a) Venn diagram of QTLs associated with growth and fat deposition and all QTLs. (b) Distribution of lincRNAs involved in QTLs associated with growth and fat deposition on chromosomes. (c) The target genes within the range of 100kb for lincRNAs corresponding to QTLs related to muscle growth and fat deposition; the figure shows the relationship between these target genes and lincRNAs. (d) The target genes within the range of 10kb for lincRNAs corresponding to QTLs related to muscle growth and fat deposition; the figure shows the relationship between these target genes and lincRNAs and the relationship between these target genes and muscle growth or fat deposition.

A total of 3,794 quantitative trait loci (QTLs) were obtained by DELs screening, involving regeneration, production, meat, carcass quality, health, and exterior (Table S6). About 18.1% (686 / 3,794) of QTLs were related with fat deposition, and 5.81% (219/ 3,794) of QTLs related to the muscle growth (Figure 5(a)). The chromosome distribution of linRNAs which were associated with muscle growth and fat deposition QTLs was shown in Figure 5(b). Even if we narrowed down all of the QTLs fragments obtained (<10kb), we still obtained some QTLs associated with muscle growth and fat deposition. After that, we continued to screen out the lincRNAs corresponding to QTLs related to muscle growth and fat deposition within 10k of the QTLs fragments and obtained a total of 7 lincRNAs related to muscle growth and fat deposition. Then, the functions of these 7 lincRNAs were predicted by analyzing the functions of corresponding adjacent genes (<100k), and the correlationships between these lincRNAs and their adjacent genes were shown in Figure 5(c). To obtain more accurate functions of lincRNAs, the range of adjacent genes of these 7 lincRNAs was then narrowed down to 10k, and the functions of these adjacent genes were statistically analyzed. These adjacent genes mainly included FGGY, GCGR, SLC25A10, PPP3CA, MRPL12, and DPYD. MRPL12 are associated with muscle growth and GCGR and SLC25A10 with fat deposition, while PPP3CA, DPYD, and FGGY are related not only to muscle growth but also to fat deposition (Figure 5(d)). Further analysis of these six target genes revealed that PPP3CA and GCGR existed in the glucagon signaling pathway and DPYD were involved in the beta-alanine metabolism pathway. This result further underscored the potential mechanisms by which differentially expressed lincRNAs are associated with muscle growth and fat deposition [36, 41].

### 3.6. Correlation Validation between lincRNAs and PTGs

Based on the expression analysis of “htseq-count,” we randomly selected 4 pairs of lincRNAs and PTGs to verify the relationship between putative lincRNAs and the corresponding PTGs by performing the qPCR experiments in corresponding tissues that included longissimus dorsi muscle, liver, and abdominal fat. All of the selected lincRNAs and their PTGs were differentially expressed, and their primer sequences for PCR experiments are shown in Table S7. First, we calculated r_0_ and p_0_ between each pair of lincRNAs and its PTGs by the cor.test() function in the R software. r_0_ and p_0_, respectively, represented the Pearson correlation coefficient and p-value of each pair of lincRNAs and its PTGs obtained in RNA-seq. Then, through the qPCR experiment, we obtained the relevant r and p, which represented the Pearson correlation coefficient and p-value of each pair of lincRNAs and its PTG calculated from the experimental data, and the biological significance of r and p is similar to that of r_0_ and p_0_, respectively. Finally, through comparison, we found that the results of 4 pairs of quantitative verifications were consistent with the trend of RNA-seq sequencing (Figures 7(a)–7(d)). 2 of the 4 lincRNAs (MSTRG.7054* vs.* GCGR, MSTRG.29576* vs.* DPYD) were randomly selected from the lincRNAs that corresponded to QTLs related to muscle growth or fat deposition within 10k of the QTL fragment. The correlations were 0.811 and 0.825, respectively, and the P-values were all less than 0.01.

## 4. Discussion

Numerous genomic studies have shown that the majority of the long noncoding RNAs in mammalian genomes are lincRNAs [17, 42]. Due to the similarity between pigs and humans in physiology process, organ development, disease research, and so on, they are widely used as important animal models, but the lincRNAs identified in pigs are far less perfect than that in humans and mice [13]. Many types of lincRNAs in pigs are still unidentified and their function remains unknown [37]. In particular, the mechanisms of the biological processes of lincRNAs underlying the regulations of the muscle growth and fat deposition still require further research. In our study, we comprehensively identified and investigated lincRNAs associated with muscle growth and fat deposition in pigs based on RNA-seq data published on NCBI.

In this study, we presented the systematical transcriptome profiling of the raw reads obtained from six samples from three tissues, including liver, abdominal fat, and longissimus dorsi muscle, using high throughput RNA-seq technology [43]. After the “fastqc” command operation, the number of clean reads obtained from the six samples was 32831240-40055406, and about 77.4% of the reads uniquely located in the reference genome, which not only further improved the gene annotation in the pig genome but also made a certain contribution to increase the number of genomes. It has been reported in the literature that most lincRNAs exhibit high tissue specificity [44, 45]. Therefore, it can be preliminarily concluded that some of these 581 putative lincRNAs are not only associated with pig muscle growth and fat deposition, but also specifically expressed. Due to people's increasing attention to pork quality, the two biological processes of muscle growth and fat deposition have gradually become the focus of later studies, and the related lincRNAs will also become the focus of research.

We characterized the identified 581 putative lincRNAs and found that they were consistent with previously reported results. Compared to protein-coding transcripts, there was shorter transcript length, smaller exon number, and longer exon length. Based on the FPKM expression levels of putative lincRNAs and protein-coding transcripts, we analyzed the specificity of liver, abdominal fat, and longissimus dorsi muscle, and then it was also demonstrated that the lincRNAs in the three tissues were more tissue-specific than the protein-coding transcripts.

To further predict whether putative lincRNAs are involved in muscle growth and fat deposition-related biological processes, we selected all DELs adjacent genes (<100 kb) for gene ontology and pathway analysis. During the analysis, we are indeed enriched with pathways and biological processes associated with muscle growth and fat deposition (Figure 4(a)). In terms of fat deposition, we have enriched the two KEGG pathways of fatty acid degradation and glucagon signaling pathway, as well as the biological processes of lipid metabolic process. Both in fatty acid degradation and glucagon signaling pathway, the CPT1A gene has been screened. In some related literature reports, the carnitine palmitoyltransferase 1 (CPT1) family gene members are necessary to transport the long-chain fatty acids into the mitochondria for oxidation. The CPT1A gene can induce mouse obesity through diet, and its expression positively correlated with BMI (R = 0.46) [46–49]. Related literature reports that PPARA that was screened together in the lipid metabolism process and the glucagon signaling pathway was a known lipid metabolism regulator [50]. In addition, PPP3CA and GCGR are also enriched in the glucagon signaling pathway. It is reported that PPP3CA is associated with intramuscular fat [51], and activation of GCGR results in decreased plasma triglyceride and cholesterol levels [52]. In terms of muscle growth, it is worth mentioning that not only do both PPP3CA and GCGR exist in the glucagon signaling pathway, but PPP3CA also exists in the skeletal muscle tissue development, and GCGR exists in the actin cytoskeleton reorganization. In addition, we have enriched two remarkable biological processes, including regulation of skeletal muscle tissue development and phospholipase C-activating G-protein coupled receptor signaling pathway and a KEGG pathway of beta-alanine metabolism. Similarly, the HOXD13 gene was simultaneously screened in both biological processes. By studying the enriched skeletal muscle tissue development, it was found that some of the target genes enriched in the pathway were significantly associated with muscle growth traits, such as TAZ, and MYL (MYL3 and MYL6) family genes. It has been reported that mutations in the TAZ gene will cause muscle weakness in mice [53]. MYL3 influence the differentiation and growth performance in quail muscle [54] and participate in the contraction of the chicken's muscles [55]. In pigs, muscle growth and muscle fiber differences are also regulated by MYL3 [56], and MYL6 was associated with fibroblast development in mice [57] (Figure 4(b)). In the phospholipase C-activating G-protein coupled receptor signaling pathway, the ESR and PTH1R were screened. It has been reported that knockout, knockdown, and overexpression of ESR will lead to overall muscle hypertrophy [58], and PTH1R accelerates myocyte differentiation [59]. In the beta-alanine metabolism, the selected DPYD gene is related not only to muscle growth but also to fat deposition. It is reported that DPYD has an effect on the longissimus muscle of Angus cattle [60], and it is related to the increase of beef marbled fat [61]. All target genes enriched in the corresponding pathways listed above are adjacent genes of DELs, and they are obviously involved in the two biological processes of muscle growth or fat deposition. So, it is speculated that some putative lincRNAs may participate in the biological processes of muscle growth and fat deposition by regulating their adjacent protein-coding genes. However, the mechanism by which individual lincRNAs regulate their adjacent genes is worthy of experimental verification and further study.

**Figure 6:**
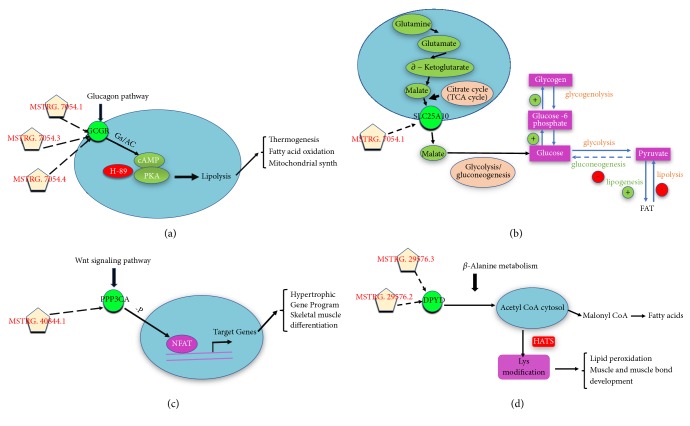
(a-d) The target genes within the range of 10kb for lincRNAs corresponding to QTLs related to muscle growth and fat deposition, and the interaction of these major target genes with the biological processes of muscle growth or fat deposition in related pathways.

**Figure 7:**
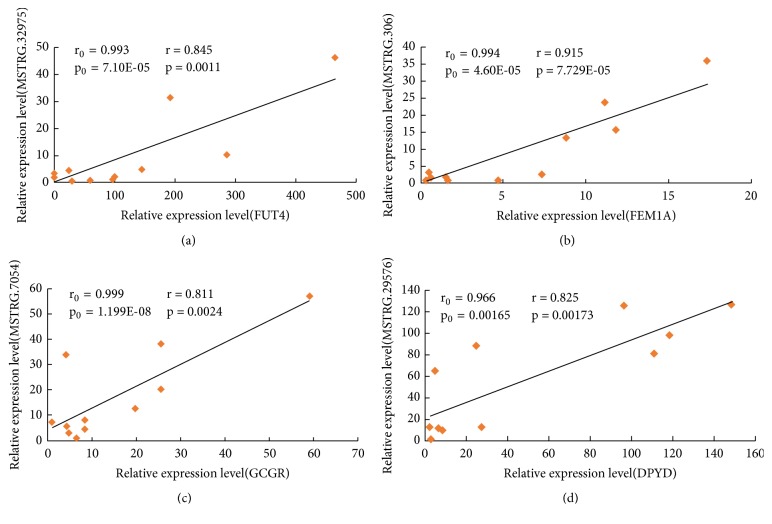
Linear regression of lincRNAs and its PTGs expression. The r_0_ and p_0_ indicate the Pearson correlation coefficient and p-value of each pair of lincRNAs and its PTGs obtained in the RNA-seq, respectively, while the r and p represent the mean in the 11 samples in quantitative verification. (a) MSTRG32975* vs.* FUT4; (b) MSTRG306* vs.* FEM1A; (c) MSTRG.7054* vs.* GCGR; (d) MSTRG.29576* vs.* DPYD.

After performing the analysis of the adjacent gene pathway of DELs, and in order to further verify the function of the putative lincRNAs, we made further functional prediction of DELs through QTLs analysis. When the range of QTLs was reduced to 10kb, we screened out 7 linRNAs corresponding to QTLs that related to muscle growth and fat deposition. Then, we screened out all the 6 adjacent genes (10kb) of 7 lincRNAs. The obtained adjacent genes were analyzed and most of them were related to muscle growth or fat deposition, which was basically consistent with our expected results. Among all 6 the adjacent genes obtained, MRPL12 is related to muscle growth. Relevant literature reports that reduced MRPL12 levels will result in overall mitochondrial translation defects in fibroblasts [62]. The genes GCGR and SLC25A10 are associated with fat deposition. It is reported that GCGR gene plays an important role in lipid metabolism [63]. At the same time, other literature reports that glucagon can bind to its receptor GCGR to regulate glucose levels and fatty acid oxidation in patients with type 2 diabetes by regulating the level of cAMPs and pka-independent pathways (Figure 6(a)) [64]. Related literature reports that the biological process by which citrate transports in exchange for malate across the mitochondrial membrane is the start of fatty acid synthesis in adipose tissue or the liver. Moreover, experiments have shown that SLC25A10 plays an important role in providing malate for citric acid transport required for fatty acid synthesis, and SiRNA was used for knockdown of SLC25A10 which is effective in reducing lipid accumulation in adipose tissue (Figure 6(b)) [65, 66]. The genes related to muscle growth and fat deposition are PPP3CA, DPYD, and FGGY. Some literature reports that calcineurin (PPP3CA) can promote the dephosphorylation of the nuclear factor of activated T cells (NFAT), allowing it to smoothly enter the nucleus from the cytoplasm, and the target gene of NFAT can participate in skeletal muscle differentiation and Hypertrophic Gene Program (Figure 6(c)) [67, 68]. The DPYD gene is involved in *β*-alanine metabolism and participated in the modification of lysine, and lysine plays an important role in lipid peroxidation and muscle and muscle bond development (Figure 6(d)). There are reports in the literature that DPYD has an effect not only on the longissimus muscle of cattle but also on the fat content of beef marble [60, 61]. FGGY gene is associated with amyotrophic lateral sclerosis [69], and FGGY mRNA is stronger in mouse WATs than in brown adipose tissue and is enhanced in gonadal fat by diet-induced obesity [70].

Both muscle and fat cells are derived from mesenchymal stem cells and are closely related during the development of the organism [71, 72]. At present, it has been found that some functional genes have a regulatory effect on both muscle and fat growth, such as PPP3CA, DPYD, and FGGY in this study. Therefore, it is possible that some related lincRNAs also regulate muscle and fat growth at the same time, which provides a good reference for the later studies of lincRNAs in muscle and fat.

Combined with pathway analysis and QTLs results, it was found that there were three of the six genes screened during QTL analysis enriched in pathways related to muscle growth or fat deposition. Both PPP3CA and GCGR are enriched in the glucagon signaling pathway, and DPYD is enriched in the beta-alanine metabolism pathway. It is also worth noting that the 7 lincRNAs and the 6 target genes in the process of QTLs analysis are almost one-to-one correspondence (Figure 5(d)). For example, only FGGY and PPP3CA are in the 10k range of MSTRG.35876.1 and MSTRG.40844.1, respectively. The different splice isoforms of MSTRG.7054 (MSTRG.7054.1, MSTRG.7054.3, and MSTRG.7054.4) all fall near the GCGR; meanwhile, the different splice isoforms of MSTRG.29576 (MSTRG.29576.3 and MSTRG.29576.2) all fall near the DPYD. Thus, we can more strongly speculate that the putative lincRNAs may participate in two biological processes of muscle growth or fat deposition by regulating its PTGs.

In this experiment, we identified and characterized all the putative lincRNAs in the liver, abdominal fat, and longissimus dorsi of pigs. After a series of analyses, especially pathway analysis and QTLs analysis, and according to quantitative verification, it was found that putative lincRNAs had a good correlation with the PTGs. Thus, we further inferred that many putative lincRNAs might be involved in muscle growth and fat deposition-related processes. Moreover, our study provides new insights into the discovery and annotation of lincRNAs related to pig muscle growth and fat deposition, especially in the analysis of differential expression of lincRNAs. Several genes are related to muscle growth and fat deposition, such as FGGY, GCGR, and PPP3CA, which are ideal candidates for later experimental verification.

## Figures and Tables

**Table 1:** Summary of data from RNA-seq.

Sample	Accession number	Raw Reads	Clean Reads	Mapping reads	Uniquely mapping ratio %
WE- liver-1	SRR167672	40,133,362	40055406	95.89	76.25
WE-liver-2	SRR167669	38,808,956	38640410	96.19	78.44
WE-af-1	SRR167673	40,000,000	39843804	96.32	78.16
WE-af-2	SRR167670	40,000,000	39865902	96.23	78.27
WE-ldm-1	SRR167674	39,164,798	34269678	96.72	76.74
WE-ldm-2	SRR167671	39,020,950	32831240	96.58	76.83

## Data Availability

The data used to support the findings of this study are available from the corresponding author upon request.

## Supplementary Materials

Supplementary Materials
* Supplementary Materials:* File S1: All lincRNAs comment files. Table S1: The specific expression of the DEGs between two tissues (af* vs.* ldm). Table S2: The specific expression of the DEGs between two tissues (af* vs.* liver). Table S3: The specific expression of the DEGs between two tissues (ldm* vs.* liver). Table S4: The basic information of the 272 DEGs. Table S5: Results of functional enrichment in gene ontology and pathway analysis. Table S6: Results of relevant QTLs screened by DELs. Table S7: PCR primers of 4 pairs of lincRNAs and PTGs.

## References

[1] Bertolini F., Schiavo G., Galimberti G. (2018). Genome-wide association studies for seven production traits highlight genomic regions useful to dissect dry-cured ham quality and production traits in Duroc heavy pigs. *Animal*.

[2] Duarte J. L., Cantet R. J., Rubio Y. L. (2016). Refining genomewide association for growth and fat deposition traits in an F pig population. *Journal of Animal Science*.

[3] Zhang J., Cui L., Ma J., Chen C., Yang B., Huang L. (2017). Transcriptome analyses reveal genes and pathways associated with fatty acid composition traits in pigs. *Animal Genetics*.

[4] Quan J., Ding R., Wang X. (2018). Genome-wide association study reveals genetic loci and candidate genes for average daily gain in Duroc pigs. *Asian-Australasian Journal of Animal Sciences*.

[5] Li P. H., Ma X., Zhang Y. Q., Zhang Q., Huang R. H. (2017). Progress in the physiological and genetic mechanisms underlying the high prolificacy of the Erhualian pig. *Yi Chuan*.

[6] Lv J., Huang Z., Liu H. (2014). Identification and characterization of long intergenic non-coding RNAs related to mouse liver development. *Molecular Genetics and Genomics*.

[7] Zou C., Li J., Luo W. (2017). Transcriptome analysis reveals long intergenic non-coding RNAs involved in skeletal muscle growth and development in pig. *Scientific Reports*.

[8] Zou C., Li L., Cheng X. (2018). Identification and functional analysis of long intergenic non-coding RNAs underlying intramuscular fat content in pigs. *Frontiers in Genetics*.

[9] Lv J., Liu H., Yu S. (2015). Identification of 4438 novel lincRNAs involved in mouse pre-implantation embryonic development. *Molecular Genetics and Genomics*.

[10] Yu H., Zhao X., Li Q. (2016). Genome-wide identification and characterization of long intergenic noncoding RNAs and their potential association with larval development in the Pacific oyster. *Scientific Reports*.

[11] Wang J., Fu L., Koganti P. P. (2016). Identification and functional prediction of large intergenic noncoding RNAs (lincRNAs) in rainbow trout (oncorhynchus mykiss). *Marine Biotechnology*.

[12] Luo H., Sun S., Li P., Bu D., Cao H., Zhao Y. (2013). Comprehensive characterization of 10,571 mouse large intergenic noncoding RNAs from whole transcriptome sequencing. *PLoS ONE*.

[13] Derrien T., Johnson R., Bussotti G. (2012). The GENCODE v7 catalog of human long noncoding RNAs: analysis of their gene structure, evolution, and expression. *Genome Research*.

[14] Khalil A. M., Guttman M., Huarte M. (2009). Many human large intergenic noncoding RNAs associate with chromatin-modifying complexes and affect gene expression. *Proceedings of the National Acadamy of Sciences of the United States of America*.

[15] Ørom U. A., Derrien T., Beringer M. (2010). Long noncoding RNAs with enhancer-like function in human cells. *Cell*.

[16] Dinger M. E., Amara P. P., Mercer T. R. (2008). Long noncoding RNAs in mouse embryonic stem cell pluripotency and differentiation. *Genome Research*.

[17] Guttman M., Garber M., Levin J. Z. (2010). Ab initio reconstruction of cell type-specific transcriptomes in mouse reveals the conserved multi-exonic structure of lincRNAs. *Nature Biotechnology*.

[18] Disteche C. M., Berletch J. B. (2015). X-chromosome inactivation and escape. *Journal of Genetics*.

[19] Zhao W., Mu Y., Ma L. (2015). Systematic identification and characterization of long intergenic non-coding RNAs in fetal porcine skeletal muscle development. *Scientific Reports*.

[20] Chen C., Ai H., Ren J. (2011). A global view of porcine transcriptome in three tissues from a full-sib pair with extreme phenotypes in growth and fat deposition by paired-end RNA sequencing. *BMC Genomics*.

[21] Xiao H., Yuan Z., Guo D. (2015). Genome-wide identification of long noncoding RNA genes and their potential association with fecundity and virulence in rice brown planthopper, Nilaparvata lugens. *BMC Genomics*.

[22] Pertea M., Kim D., Pertea G. M., Leek J. T., Salzberg S. L. (2016). Transcript-level expression analysis of RNA-seq experiments with HISAT, StringTie and Ballgown. *Nature Protocols*.

[23] Zou C., Li S., Deng L. (2017). Transcriptome analysis reveals long intergenic noncoding RNAs contributed to growth and meat quality differences between yorkshire and wannanhua pig. *Genes-Basel*.

[24] Kong L., Zhang Y., Ye Z.-Q. (2007). CPC: assess the protein-coding potential of transcripts using sequence features and support vector machine. *Nucleic Acids Research*.

[25] Pirooznia M., Perkins E. J., Deng Y. (2008). Batch Blast Extractor: An automated blastx parser application. *BMC Genomics*.

[26] Robich M. P., Osipov R. M., Chu L. M. (2010). Temporal and spatial changes in collateral formation and function during chronic myocardial ischemia. *Journal of the American College of Surgeons*.

[27] Love M. I., Huber W., Anders S. (2014). Moderated estimation of fold change and dispersion for RNA-seq data with DESeq2. *Genome Biology*.

[28] Zhang G., Chen D., Zhang T., Duan A., Zhang J., He C. (2018). Transcriptomic and functional analyses unveil the role of long non-coding RNAs in anthocyanin biosynthesis during sea buckthorn fruit ripening. *DNA Research*.

[29] Guttman M., Amit I., Garber M. (2009). Chromatin signature reveals over a thousand highly conserved large non-coding RNAs in mammals. *Nature*.

[30] Ponjavic J., Oliver P. L., Lunter G., Ponting C. P. (2009). Genomic and transcriptional co-localization of protein-coding and long non-coding RNA pairs in the developing brain. *PLoS Genetics*.

[31] Cabili M. N., Trapnell C., Goff L. (2011). Integrative annotation of human large intergenic noncoding RNAs reveals global properties and specific subclasses. *Genes & Development*.

[32] Salleh F. H. M., Arif S. M., Zainudin S., Firdaus-Raih M. (2015). Reconstructing gene regulatory networks from knock-out data using gaussian noise model and pearson correlation coefficient. *Computational Biology and Chemistry*.

[33] Quinlan A. R., Hall I. M. (2010). BEDTools: a flexible suite of utilities for comparing genomic features. *Bioinformatics*.

[34] Benjamini Y., Drai D., Elmer G., Kafkafi N., Golani I. (2001). Controlling the false discovery rate in behavior genetics research. *Behavioural Brain Research*.

[35] Liao Q., Liu C., Yuan X. (2011). Large-scale prediction of long non-coding RNA functions in a coding-non-coding gene co-expression network. *Nucleic Acids Research*.

[36] Zhao P., Zheng X., Feng W. (2018). Profiling long noncoding RNA of multi-tissue transcriptome enhances porcine noncoding genome annotation. *Epigenomics*.

[37] Li J., Gao Z., Wang X., Liu H., Zhang Y., Liu Z. (2016). Identification and functional analysis of long intergenic noncoding RNA genes in porcine pre-implantation embryonic development. *Scientific Reports*.

[38] Tang Z., Wu Y., Yang Y. (2017). Comprehensive analysis of long non-coding RNAs highlights their spatio-temporal expression patterns and evolutional conservation in Sus scrofa. *Scientific Reports*.

[39] Liu S., Zhang Y., Wang X. (2017). Annotation and cluster analysis of spatiotemporaland sex-related lncRNA expression in rhesus macaque brain. *Genome Research*.

[40] Casero D., Sandoval S., Seet C. S. (2015). Long non-coding RNA profiling of human lymphoid progenitor cells reveals transcriptional divergence of B cell and T cell lineages. *Nature Immunology*.

[41] Long Y., Ruan G. R., Su Y. (2015). Genome-wide association study identifies QTLs for EBV of backfat thickness and average daily gain in Duroc pigs. *Genetika*.

[42] Hartig S. M., He B., Long W., Buehrer B. M., Mancini M. A. (2011). Homeostatic levels of SRC-2 and SRC-3 promote early human adipogenesis. *The Journal of Cell Biology*.

[43] Qian X., Ba Y., Zhuang Q., Zhong G. (2014). RNA-seq technology and its application in fish transcriptomics. *OMICS: A Journal of Integrative Biology*.

[44] Washietl S., Kellis M., Garber M. (2014). Evolutionary dynamics and tissue specificity of human long noncoding RNAs in six mammals. *Genome Research*.

[45] Amin V., Harris R. A., Onuchic V. (2015). Epigenomic footprints across 111 reference epigenomes reveal tissue-specific epigenetic regulation of lincRNAs. *Nature Communications*.

[46] Warfel J. D., Vandanmagsar B., Dubuisson O. S. (2017). Examination of carnitine palmitoyltransferase 1 abundance in white adipose tissue: Implications in obesity research. *American Journal of Physiology-Regulatory, Integrative and Comparative Physiology*.

[47] Rameshreddy P., Uddandrao V. V. S., Brahmanaidu P. (2018). Obesity-alleviating potential of asiatic acid and its effects on ACC1, UCP2, and CPT1 mRNA expression in high fat diet-induced obese Sprague–Dawley rats. *Molecular and Cellular Biochemistry*.

[48] Skotte L., Koch A., Yakimov V. (2017). CPT1A Missense Mutation Associated with Fatty Acid Metabolism and Reduced Height in Greenlanders. *Circulation: Cardiovascular Genetics*.

[49] Calderon-Dominguez M., Sebastián D., Fucho R. (2016). Carnitine palmitoyltransferase 1 increases lipolysis, UCP1 protein expression and mitochondrial activity in brown adipocytes. *PLoS ONE*.

[50] Pearsall E. A., Cheng R., Zhou K. (2017). PPAR*α* is essential for retinal lipid metabolism and neuronal survival. *BMC Biology*.

[51] Davoli R., Luise D., Mingazzini V. (2016). Genome-wide study on intramuscular fat in Italian Large White pig breed using the PorcineSNP60 BeadChip. *Journal of Animal Breeding and Genetics*.

[52] More V. R., Lao J., McLaren D. G. (2017). Glucagon like receptor 1/ glucagon dual agonist acutely enhanced hepatic lipid clearance and suppressed de novo lipogenesis in mice. *PLoS ONE*.

[53] Johnson J. M., Ferrara P. J., Verkerke A. R. P. (2018). Targeted overexpression of catalase to mitochondria does not prevent cardioskeletal myopathy in Barth syndrome. *Journal of Molecular and Cellular Cardiology*.

[54] Park J.-W., Lee J. H., Kim S. W. (2018). Muscle differentiation induced up-regulation of calcium-related gene expression in quail myoblasts. *Asian-Australasian Journal of Animal Sciences*.

[55] Ouyang H., Wang Z., Chen X., Yu J., Li Z., Nie Q. (2017). Proteomic analysis of chicken skeletal muscle during embryonic development. *Frontiers in Physiology*.

[56] Wang Z., Shang P., Li Q. (2017). iTRAQ-based proteomic analysis reveals key proteins affecting muscle growth and lipid deposition in pigs. *Scientific Reports*.

[57] Eichenmüller M., Bauer R., Von Schweinitz D., Hahn H., Kappler R. (2007). Hedgehog-independent overexpression of transforming growth factor-beta1 in rhabdomyosarcoma of Patched1 mutant mice. *International Journal of Oncology*.

[58] Verbrugge S. A. J., Schönfelder M., Becker L., Nezhad F. Y., de Angelis M. H., Wackerhage H. (2018). Genes whose gain or loss-of-function increases skeletal muscle mass in mice: A systematic literature review. *Frontiers in Physiology*.

[59] Kimura S., Yoshioka K. (2014). Parathyroid hormone and parathyroid hormone type-1 receptor accelerate myocyte differentiation. *Scientific Reports*.

[60] Santana M. H. A., Ventura R. V., Utsunomiya Y. T. (2015). A genomewide association mapping study using ultrasound-scanned information identifies potential genomic regions and candidate genes affecting carcass traits in Nellore cattle. *Journal of Animal Breeding and Genetics*.

[61] Lim D., Kim N.-K., Lee S. H. (2014). Characterization of genes for beef marbling based on applying gene coexpression network. *International Journal of Genomics*.

[62] Serre V., Rozanska A., Beinat M. (2013). Mutations in mitochondrial ribosomal protein MRPL12 leads to growth retardation, neurological deterioration and mitochondrial translation deficiency. *Biochimica et Biophysica Acta (BBA) - Molecular Basis of Disease*.

[63] Gu W., Lloyd D. J., Chinookswong N. (2011). Pharmacological targeting of glucagon and glucagon-like peptide 1 receptors has different effects on energy state and glucose homeostasis in diet-induced obese mice. *The Journal of Pharmacology and Experimental Therapeutics*.

[64] Li X., Zhuo J. L. (2007). Targeting glucagon receptor signalling in treating metabolic syndrome and renal injury in type 2 diabetes: theory versus promise. *Clinical Science*.

[65] Mizuarai S., Miki S., Araki H., Takahashi K., Kotani H. (2005). Identification of dicarboxylate carrier Slc25a10 as malate transporter in de Novo fatty acid synthesis. *The Journal of Biological Chemistry*.

[66] Kulyte A., Ehrlund A., Arner P., Dahlman I. (2017). Global transcriptome profiling identifies KLF15 and SLC25A10 as modifiers of adipocytes insulin sensitivity in obese women. *PLoS ONE*.

[67] Jingting S., Qin X., Yanju S. (2017). Oxidative and glycolytic skeletal muscles show marked differences in gene expression profile in Chinese Qingyuan partridge chickens. *PLoS ONE*.

[68] Xia J., Zhang Y., Xin L. (2015). Global transcriptomic profiling of cardiac hypertrophy and fatty heart induced by long-term high-energy diet in bama miniature pigs. *PLoS ONE*.

[69] Daoud H., Valdmanis P. N., Dion P. A., Rouleau G. A. (2010). Analysis of DPP6 and FGGY as candidate genes for amyotrophic lateral sclerosis. *Amyotrophic Lateral Sclerosis and Frontotemporal Degeneration*.

[70] Taylor J. A., Shioda K., Mitsunaga S. (2018). Prenatal exposure to bisphenol a disrupts naturally occurring bimodal DNA methylation at proximal promoter of fggy, an obesity-relevant gene encoding a carbohydrate kinase, in gonadal white adipose tissues of CD-1 Mice. *Endocrinology*.

[71] Jeong B., Kang I., Hwang Y., Kim S., Koh J. (2014). MicroRNA-194 reciprocally stimulates osteogenesis and inhibits adipogenesis via regulating COUP-TFII expression. *Cell Death & Disease*.

[72] Divoux A., Karastergiou K., Xie H. (2014). Identification of a novel lncRNA in gluteal adipose tissue and evidence for its positive effect on preadipocyte differentiation. *Obesity*.

